# Rapid identification of staphylococci by Raman spectroscopy

**DOI:** 10.1038/s41598-017-13940-w

**Published:** 2017-11-01

**Authors:** Katarína Rebrošová, Martin Šiler, Ota Samek, Filip Růžička, Silvie Bernatová, Veronika Holá, Jan Ježek, Pavel Zemánek, Jana Sokolová, Petr Petráš

**Affiliations:** 1 Department of Microbiology, Faculty of Medicine, Masaryk University and St. Anne’s Faculty Hospital, Pekařská 53, Brno, 65691 Czech Republic; 2 Institute of Scientific Instruments of the Czech Academy of Sciences, v.v.i., Královopolská 147, Brno, 61264 Czech Republic; 3 National Reference Laboratory for Staphylococci, National Institute of Public Health, Šrobárova 48, Praha, 100 42 Czech Republic

## Abstract

Clinical treatment of the infections caused by various staphylococcal species differ depending on the actual cause of infection. Therefore, it is necessary to develop a fast and reliable method for identification of staphylococci. Raman spectroscopy is an optical method used in multiple scientific fields. Recent studies showed that the method has a potential for use in microbiological research, too. Our work here shows a possibility to identify staphylococci by Raman spectroscopy. We present a method that enables almost 100% successful identification of 16 of the clinically most important staphylococcal species directly from bacterial colonies grown on a Mueller-Hinton agar plate. We obtained characteristic Raman spectra of 277 staphylococcal strains belonging to 16 species from a 24-hour culture of each strain grown on the Mueller-Hinton agar plate using the Raman instrument. The results show that it is possible to distinguish among the tested species using Raman spectroscopy and therefore it has a great potential for use in routine clinical diagnostics.

## Introduction

Staphylococci are gram-positive bacteria inhabiting the skin and mucosal membranes of humans^1,2^. However, under certain circumstances (disruption of skin, diminution of immunity), they can cause infections of variable severity^3^. The most commonly found staphylococcal species in clinical material is *Staphylococcus aureus*. This pathogen can produce a broad range of virulence factors—haemolysins, proteases, leukocidins, toxic shock syndrome toxin, exfoliative toxins, enterotoxins plus immune-modulatory factors^4^—and can cause mild to severe infections including infections of skin and soft tissues, toxin-mediated food poisoning and toxic shock syndrome. After entering the bloodstream, *S*. *aureus* may become the cause of sepsis, endocarditis, osteomyelitis, meningitis or other life-threatening invasive diseases^5^. These infections are often hospital-acquired^6^ and caused by multiresistant strains^6,7^. Another coagulase-positive staphylococci that can rarely cause infections in humans (meningitidis, skin absceses) belong to the *Staphylococcus intermedius* group^8,9^.

Coagulase-negative staphylococci are, in contrast to *S*. *aureus*, saprophytic organisms living on human skin and are often found in clinical material as contaminants. However, in the recent years, they significantly contributed to the ever-increasing morbidity and mortality of nosocomial infections^10^. The majority of infections caused by coagulase-negative staphylococci are associated with biofilm formation that can occur on artificial materials and/or medical devices in the human body as well as on disrupted tissues of the patient^2,11–16^. These infections are difficult to treat and can be fatal. The most frequently isolated species from clinical specimens is *Staphylococcus epidermidis* ^2^. Other clinically important species that can be found in human clinical material include *Staphylococcus haemolyticus* (bloodstream infections, endocarditis, peritonitis and foreign-body infections)^17–20^, *Staphylococcus lugdunensis* and *Staphylococcus saprophyticus* (arthritis, urinary tract infections)^17,21,22^, *Staphylococcus hominis*, *Staphylococcus caprae*, *Staphylococcus warneri*, *Staphylococcus capitis*, *Staphylococcus schleiferi*, *Staphylococcus xylosus* and *Staphylococcus auricularis* (various infections)^17,23^, *Staphylococcus sciuri* (wounds, endocarditis)^17,24,25^, *Staphylococcus simulans* (cutaneous infections, osteoarticular infections, endocarditis)^26^ and *Staphylococcus petrasii* (ear infections)^27^.

As the character of infections caused by *Staphylococcus aureus* and coagulase-negative staphylococci differ, it is important to distinguish between these two groups and subsequently adjust the treatment. Therefore we tested the possibility to use a fast, relatively cheap, contactless, label-free spectroscopic method—Raman spectroscopy—for identification of the clinically most relevant staphylococcal species. In our comprehensive study on bacteria we have included 16 clinically relevant strains, with 277 sub-strains in order to cover most of the family of staphylococci. This could lead to the generation of a large reference database/library for Raman spectra of bacteria to unambiguously determine the identity of an unknown bacterial sample. Please note that our Raman spectral library presented here has been directed towards the clinical needs of the Department of Microbiology at St. Anne’s Faculty Hospital in Brno and can also be used as a starting point by other groups involved in clinical applications.

Raman spectroscopy is based on the shift between the frequency of incident and scattered light that is called the Raman effect. This shift is induced by molecular vibrations in the sample^28,29^ and makes Raman spectroscopy an useful tool for identification and characterization of biological systems^30–50^.

## Materials and Methods

### Microorganisms and sample preparation

The experiments included 277 staphylococcal strains: 62 *Staphylococcus aureus* strains, 8 *Staphylococcus intermedius/pseudointermedius* strains and 207 strains of coagulase-negative staphylococci (63 strains of *Staphylococcus epidermidis*, 21 strains of *Staphylococcus haemolyticus*, 21 strains of *Staphylococcus hominis*, 16 strains of *Staphylococcus petrasii*, 15 strains of *Staphylococcus saprophyticus*, 11 strains of *Staphylococcus lugdunensis*, 11 strains of *Staphylococcus warneri*, 9 strains of *Staphylococcus sciuri*, 8 strains of *Staphylococcus schleiferi*, 7 strains of *Staphylococcus capitis*, 7 strains of *Staphylococcus xylosus*, 6 strains of *Staphylococcus auricularis*, 6 strains of *Staphylococcus caprae* and 6 strains of *Staphylococcus simulans*). The majority of strains, excluding *S*. *aureus* and *S*. *epidermidis* strains, were kindly provided by Petr Petráš from the National Reference Laboratory for Staphylococci in Prague, Czech Republic. Furthermore, the set of analysed strains included eighteen reference strains (*S*. *aureus* CCM 7111, CCM 3953, CCM 4750 and CCM 4223, *S*. *epidermidis* CCM 7221 and CCM 2124, *S*. *hominis* CCM 2732, *S*. *haemolyticus* CCM 2729, *S*. *capitis* subsp. *capitis* CCM 2735, *S*. *lugdunensis* CCM 4068, *S*. *caprae* CCM 4546, *S*. *schleiferi* subsp. *schleiferi* CCM 4070, *S*. *sciuri* subsp. *sciuri* CCM 7040, *S*. *saprophyticus* subsp. *saprophyticus* CCM 2727, *S*. *simulans* CCM 2724, *S*. *xylosus* CCM 2725, *S*. *warneri* CCM 2731, *S*. *intermedius* CCM 4710) from the Czech Collection of Microorganisms (CCM) and one reference strain from the Czech National Collection of Type Cultures (CNCTC) – *S*. *aureus* CNCTC 7452. The other strains were clinical isolates stored in the Culture Collection of the Department of Microbiology, St. Anne’s Faculty Hospital in Brno, Czech Republic. All of the strains were identified using biochemical methods plus MALDI-TOF mass spectrometry and stored at −70 °C.

For the purpose of this experiment, the strains were thawed, inoculated onto Mueller-Hinton agar plates (MH, Oxoid, Basingstoke, United Kingdom) and cultivated for 24 hours at 37 °C. These conditions were selected in accordance with our previous work^51^.

### Experimental setup

After cultivation, staphylococcal colonies were measured by a commercial Renishaw Raman spectrometer (Renishaw inVia Raman Spectrometer, Renishaw plc., Wotton-under-Edge, UK). Measurement settings were the same as described in our previous work^51^. Briefly, laser: single-mode diode, wavelength: 785 nm, microscope objective: Leica, Wetzlar, Germany, with numerical aperture 0.5, magnification: 500x, laser spot dimensions: approximately 2 μm × 10 μm, working distance: 0.5 mm, minimal number of measurements/strain: 10 (from at least 3 different bacterial colonies), spectral acquisition: 15 seconds. Each spectrum consists of 1015 points measured in the range 614–1724 cm^−1^.

The geometry described above ensures that the Raman signal is collected over an axial range of about 8 µm and, therefore, a contribution from cultivation media to the Raman spectra can be neglected^51^. Before each spectral acquisition, the laser was refocused onto a colony surface ensuring that the collected signal originates within the focal depth of the laser excitation and imaging optics.

On one day we acquired maximum of 200 Raman spectra. Measurements were performed in the same way on all measurement days. This applies also for the sample preparation. Reproducibility of microbial Raman spectra acquired this way was validated in our previous work^51^.

### Data analysis

Raman spectroscopy typically suffers from a strong fluorescence background. Such background can be typically removed by various mathematical techniques^52,53^, such as polynomial baseline fitting, consequent numerical differentiation and integration, etc. It is even possible to suppress the fluorescence directly in the experiment using the frequency modulation of exciting laser^54^. Each method slightly disturbs the original spectrum and, therefore, may influence the quality of bacterial strain identification. We used two methods, namely Rolling-Circle Filter (RCF)^55^ (10 passes, 350 points circle radius) and iterative polynomial fitting (IPF)^52^ (maximum of 10 passes, 12^th^ polynomial order). Subsequently, high-frequency noise was removed using Savistky-Golay filtering (2^nd^ order, width 7points) and the spectra are normalized to the area of phenylalanine peak in the wavenumber range of 996–1009 cm^−1^ ^39^.

Prior the spectral based identification we employed Principal Component Analysis (PCA) in order to extract the main features of the spectra and then use these features for bacterial strain identification. The whole ensemble of spectra is described by so-called loadings and principal component (PC) scores. The loadings represent an orthonormal coordinate base having the same dimension as is the number of measured Raman shifts and scores correspond to “coordinates” in this space. The PCA selects the loadings in a way that maximal variance of the original data is described by the first several scores. Therefore, it is typically sufficient to take into account only first 10–20 PC scores that would almost completely characterise the whole spectrum instead of the 1015 values corresponding to the intensities of each Raman shift.

In order to identify bacterial strains based on their Raman spectrum we used and compared 3 three methods typically used in computer science, namely Linear Discriminant Analysis (LDA)^56^, one nearest neighbor (1NN)^57^ and Support Vector Machine (SVM)^58^. These methods are often used in the field of computer vision or machine learning and were already used for Raman spectra identification^53,59–63^, too. They belong to the large group of supervised learning models, where the methods are initially “trained” using already known results.

In order to evaluate the performance of identification of staphylococci we used 5-fold cross-validation, i.e. the measured data set was randomly separated into 5 equally sized groups, the classification methods were trained using 80% of data and then the remaining 20% of data was classified using the trained model. For the identification we used MATLAB (Mathworks Inc., Natick, MA, USA) functions *fitcdiscr*, *fitcknn*, and *fitcecoc* (part of the Statistics and Machine Learning Toolbox of MATLAB). Furthermore, we optimised the number of PC scores used for bacterial identification in the range of 2–50.

### Data Availability

The datasets generated during and/or analysed during the current study are available from the corresponding author on reasonable request.

## Results and Discussion

We collected Raman fingerprints from 277 staphylococcal strains (70 of coagulase-positive and 207 of coagulase-negative).

**Figure 1:**
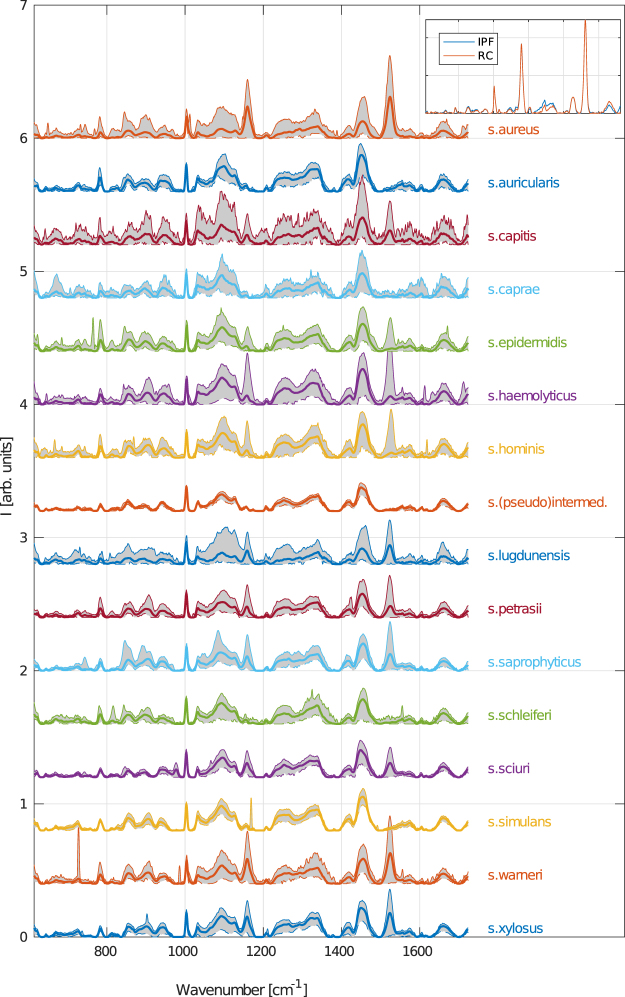
Averaged Raman spectra of all measured staphylococcal species (thick curves). The grayed area depicts the variations of measured spectral intensities corresponding to a given wavenumber. Border curves of this interval correpond to 0.1^st^ (dashed) and 99.9^th^ percentiles, respectively. Fluorescence spectral background was removed by the IPF method. The top-right inset compares both background removal methods (IPF and RC) on a single randomly selected spectrum of *S*. *aureus*.

The averaged Raman spectra of each bacterial species (thick curves) are shown in Fig. 1. The fluorescent background was removed by the IPF method. The variance of spectra is marked by the gray area that borders 0,1^st^ and 99,9^th^ percentile of spectral intensity variations for a given Raman shift. Please note, that we used the percentiles instead of error bands based on stadard deviation of data since the measred spectral intensities are not normally distribured. The selected percentiles would correspond to commonly used 3σ interval for normally distributed data.

The comparison of background removal methods can be seen in the inset of Fig. 1. A single, randomly selected spectrum of a *S*. *aureus* species is shown after filtering based on both IPF as well as RC background removal methods. Certain variations of less prominent spectral band might be seen but the positions and intensities of the most characteristic peaks are conserved.

The narrow phenylalalnine peak (at 1005 cm^−1^) is clearly present in all spectra and thus it is reasonable to use it as an internal standard for normalization. Furthermore, we observe quite strong variation of spectra even within one staphycoccal species which is depicted the separation of gray curves. One of the distinctive features is the presence or absence of peaks connected to carotenoids vibration in the wavenumber ranges 1110, 1160 and 1525 cm^−1^ corresponding to C-C-(CH3), =C-C=, and -C=C vibrations^64^. We see that certain species in our work do not exhibit those at all (such as *Staphylococcus caprae*, *Staphylococcus* (*pseudo*)*intermedius*, *Staphylococcus schleiferi* and *Staphylococcus simulans*) while the other strains exhibit medium or strong carotenoid signals. This is especially the case for *Staphylococcus aureus*, even though the carotenoid of this species varies strongly. Another feature noticable by naked eye is that *Staphylococcus sciuri* is the only species exhibiting peaks at 977 cm^−1^.

**Figure 2:**
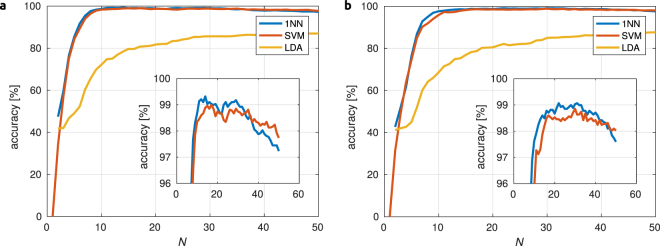
Accuracy of staphycoccal strain identification for all three used methods (1 nearest neighbor, support vector machines and linear discriminant analysis) as a function of the number of used PCA scores *N*. Fluorescence background was removed using iterative polynomial fitting (**a**) or rolling circle (**b**) methods. Insets show magnified regions, with accuracy above 96%.

For identification of staphylococcal strains we used three methods: Linear Discriminant Analysis (LDA), one nearest neighbor (1NN) and Support Vector Machine (SVM). The accuracy of identification, i.e. relative count of correctly identified samples, depends both on the fluorescence background subtraction method as well as on the number of PC scores *N* considered for identification. Figure 2 shows the accuracy as a function of *N* used for identification for both IPF and RC background removal methods. One can see that 1NN and SVM exceed 98% accuracy for 10 PC scores and stay above this value up to *N*~30. Slightly better results are obtained for the IPF background removal method. Table 1 summarizes the best achieved results for each identification method as well as for both background removal methods.

**Table 1:** Performance of staphycoccal strain identification using three algorithms (LDA, 1NN, and SVM) for $2\,=$ two methods of fluorescence background removal (IPF and RCF).

Identification Method	Background removal method			
	IPF		RCF	
	accuracy [%]	*N*_opt_	accuracy [%]	*N*_opt_
LDA	87.1	47	87.3	49
1NN	99.3	14	99.2−	24
SVM	98.8	14	98.9	27

Numbers in table cells correspond to the accuracy of identification, i.e. percentage of successful identification upon using 5-fold verification scheme, and values of *N*_opt_ give the number of PC scores used for such a successful identification. Abbreviations: LDA = Linear Discriminant Analysis, 1NN = One Nearest Neighbor, SVM = Support Vector Machine, IPF = Iterative Polynomial Fitting, RCF = Rolling-Circle Filter.

We can see that the highest level of identification is obtained using the 1NN algorithm that is applied on 14 PC scores and with the fluorescence background removal using IPF. Both 1NN and SVM give total successful identification around 99% with slightly better results for IPF background removal. LDA identification gives an accuracy of only around 87%.

**Figure 3:**
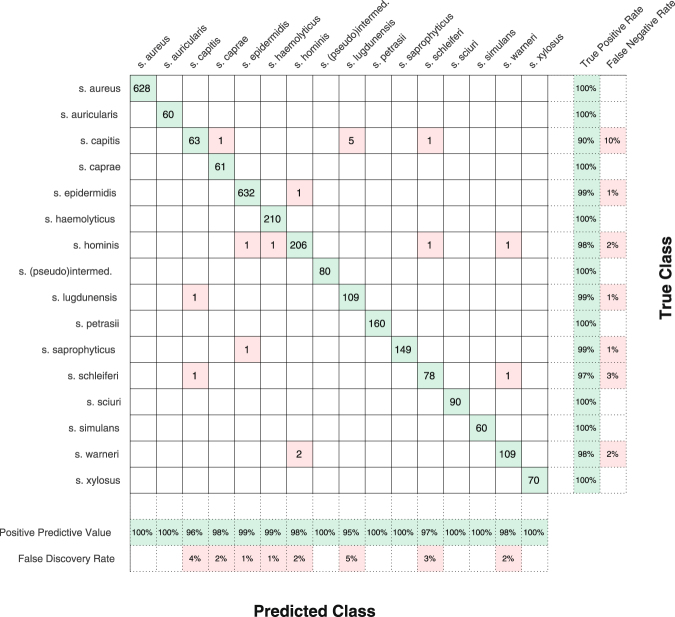
Confusion matrix showing the result of 5-fold cross-validated bacterial strain identification. Each row of the main part of table corresponds to bacterial species identified by MALDI-TOF MS plus biochemical methods (True Class) and each column corresponds to the bacterial identification predicted by the 1 nearest neighbor algorithm algorithm that employed iterative polynomial fitting background fluorescence removal and 14 PC scores. Numbers in the cells stand for correctly classified (diagonal) or missclassified (off-diagonal, red) spectra, respectively. The two rightmost columns show the sensitivity (True Positive Rate) and False Negative Rate), while two bottom columns show the Positive Predictive Value and the False Discovery Rate (values are rounded to integer values).

Figure 3 shows the results of individual staphycoccal species identification in the form of the Confusion matrix^65^. We used the best approach shown in Table 1, i.e. the 1NN method, IPF background removal, and *N* = 14. Rows and columns of the Confusion matrix correspond to species identification by MALDI-TOF MS accompanied by biochemical testing (denoted as True Class) and species identification using Raman spectra (Predicted Class), respectively. The total number of correctly identified spectra is shown on the diagonal while the number of incorrectly identified strains is placed off-diagonal in a grayed background. These off-diagonal elements show how the spectra of a certain strains are mis-assigned to a different species. Furthermore, the rightmost columns show the sensitivity (True Positive Rate) and False Negative Rate), i.e. the relative count of Raman spectra measured from the given strains that was correctly or incorrectly identified using the 1NN method.

Similarly, the two bottom rows show the Positive Predictive Value and the False Discovery Rate, i.e. the relative count of properly identified spectra and spectra corresponding to different species that were identified as the one in the given column. Ideally, both True Positive Rate as well as Positive Predictive Value should be 100%. This was achieved for *S*. *aureus*, *S*. *auricularis*, *S*. (*pseudo*)*intermedius*, *S*.*petrasii*, *S*. *sciuri*, *S*. *simulans* and *S*. *xylosus*.

*S*. *saprophyticus* has a 100% Positive Predictive Value and a nearly non-zero False Negative Rate, i.e. no other spectra were identified as *S*. *saprophyticus* but one spectrum of this species (out of 150) was identified as *S*. *epidermidis*.

100% True Positive Rate and non-zero False Discovery Rate mean that all spectra of given species were identified correctly, but that some spectra of other species were incorrectly identified as the given species. This happened for *S*. *caprae* and *S*. *heamolyticus*. Probably the worst identification was obtained for *S*. *capitis*. However, both the False Negative Rate and the False Discovery Rate were still only 10 and 5 percent, respectively.

Even better results are achieved if we do not consider individual spectra but we look at the individual strains (10 spectra/strain). With this approach we get the overall success rate of 100%.

Moreover, we tested the performance of spectral identification on a reduced sample set without cross-validaton. Firstly, we randomly excluded approximately 20% of staphylococcal strains from the total set of measured spectra. We excluded at least one strain per species for identification of previously unknown strains. Consenquently, PCA was performed on the reduced spectral set and both the 1NN and SVN classificators were trained using 14 PC scores. In the next step, the excluded spectra were transformed into PC scores (using previously obtained loadings) that were identified using both methods. Depending on the seclection of excluded strains we achieved sucessfull identification of individual spectra in the range of 70–80%. However, if we consider only the strains that were completly mis-assigned to a single incorrect species by both methods we obtain that only up to 4% of excluded strains are completly mis-assigned. Thus, in this case we achieved also a very high accuracy of about 96%.

Our results show that it is possible to efficiently identify staphylococci using Raman spectroscopy. This is in good agreement with previous studies finding similar results for the identification of different species^30,33,44,46,47^. Recent studies also suggest that we can use Raman spectroscopy for the detection of antimicrobial resistance^48,66^ and other virulence factors like the ability to form biofilms^37,41,43^. These studies support the prospective use of Raman spectroscopy as a tool for microbial diagnostics. Moreover, the Raman fingerprints are highly reproducible. This was already proven for yeast spectra acquired in a time window of more than one year and the findings were supported by our recent work on *S*. *aureus* and *S*. *epidermidis* ^38,51^.

The high reproducibility coupled with the diagnostic potential presented here suggest the high potential of Raman spectroscopy for clinical diagnostics and that further research in this field is very promising. It is supported by the advantages of Raman spectroscopy including speed, low cost analyses and non-destructive nature. The non-destructive nature of the method allows for use of the sample in subsequent analyses. Raman spectroscopy does not require any time-consuming sample preparation nor the use of additional chemicals or materials, which is common for other routinely used biochemical methods or the commonly used MALDI-TOF mass spectroscopy.

Current disadvantages of Raman spectroscopy as a diagnostic tool include the absence of commercial databases for identification of bacterial spectra. This suggests the need for further investigations in this field that will help to build an automated diagnostic tool in the future. Also, in order to make a comparison of the spectra measured on our system with those measured on different Raman instruments we should consider that the quantum efficiency of the given detector and optical elements are wavelength dependent. This suggests that the data should be corrected according to the instrument response profile. In an ideal case the spectral sensitivity curve should be used so that the raw data from different systems can be transferred/evaluated. In many cases it is difficult to obtain the instrument response from the transmission and/or reflectivity of all optical elements in different Raman systems. Thus, we have corrected our data only for the quantum efficiency of the detector which can be readily obtained from the manufacturer. In the next step we used this corrected data for the analysis describe above to see any influence on the final data – we obtained the same results with only minor redistribution of off-diagonal elements in the confusion matrix. Also, the repeatability of our data collection has been proved by spectral identification on a reduced sample set with/without cross-validaton detailed in this section. However, we would like to note that for our “library” (developed for the Department of Microbiology at St. Anne’s Faculty Hospital in Brno) to be compared with spectra measured on different instruments the spectral sensitivity curves of given instruments should be provided. This can be obtained from a manufacturer on request.

Since Raman spectroscopy can also be used for the identification of certain virulence factors and antimicrobial resistance, it could make this method a very useful diagnostic tool providing a wide spectrum of characteristic information, in addition to strain identification, in one single measurement. That might significantly accelerate the diagnostic process.

In conclusion, analyses of Raman spectra acquired from 277 staphylococcal strains belonging to 16 species suggest that Raman spectroscopy can be used as a reliable tool for identification of staphylococci. We were able to achieve the total success rate of more than 99% for individual spectra and even 100% for a few individual strains.

### Summary

The goal of the article was to assess Raman spectroscopy as a potential method for identification of 16 clinically important staphylococcal species since this method could accelerate the diagnostic process and lower the costs both for diagnostics and consequently treatment of patients.
